# Inhibition of Ionic Currents by Fluoxetine in Vestibular Calyces in Different Epithelial Loci

**DOI:** 10.3390/ijms25168801

**Published:** 2024-08-13

**Authors:** Nesrien M. M. Mohamed, Frances L. Meredith, Katherine J. Rennie

**Affiliations:** Department of Otolaryngology-Head & Neck Surgery, University of Colorado School of Medicine, Aurora, CO 80045, USA; nesrien.mohamed@cuanschutz.edu (N.M.M.M.); frances@meredith-leroux.org (F.L.M.)

**Keywords:** vestibular calyx, fluoxetine, vestibular hair cell, semicircular canal, Na^+^ channel, K^+^ channel

## Abstract

Previous studies have suggested a role for selective serotonin reuptake inhibitors (SSRIs) such as fluoxetine (Prozac^®^) in the treatment of dizziness and inner ear vestibular dysfunction. The potential mechanism of action within the vestibular system remains unclear; however, fluoxetine has been reported to block certain types of K^+^ channel in other systems. Here, we investigated the direct actions of fluoxetine on membrane currents in presynaptic hair cells and postsynaptic calyx afferents of the gerbil peripheral vestibular system using whole cell patch clamp recordings in crista slices. We explored differences in K^+^ currents in peripheral zone (PZ) and central zone (CZ) calyces of the crista and their response to fluoxetine application. Outward K^+^ currents in PZ calyces showed greater inactivation at depolarized membrane potentials compared to CZ calyces. The application of 100 μM fluoxetine notably reduced K^+^ currents in calyx terminals within both zones of the crista, and the remaining currents exhibited distinct traits. In PZ cells, fluoxetine inhibited a non-inactivating K^+^ current and revealed a rapidly activating and inactivating K^+^ current, which was sensitive to blocking by 4-aminopyridine. This was in contrast to CZ calyces, where low-voltage-activated and non-inactivating K^+^ currents persisted following application of 100 μM fluoxetine. Additionally, marked inhibition of transient inward Na^+^ currents by fluoxetine was observed in calyces from both crista zones. Different concentrations of fluoxetine were tested, and the EC_50_ values were found to be 40 µM and 32 µM for K^+^ and Na^+^ currents, respectively. In contrast, 100 μM fluoxetine had no impact on voltage-dependent K^+^ currents in mechanosensory type I and type II vestibular hair cells. In summary, micromolar concentrations of fluoxetine are expected to strongly reduce both Na^+^ and K^+^ conductance in afferent neurons of the peripheral vestibular system in vivo. This would lead to inhibition of action potential firing in vestibular sensory neurons and has therapeutic implications for disorders of balance.

## 1. Introduction

Dizziness is a common complaint encountered in medical practice, but its underlying cause is often unclear. Previous studies have suggested that selective serotonin reuptake inhibitors (SSRIs) such as fluoxetine (Prozac^®^) might help alleviate dizziness and inner ear vestibular dysfunction [1]. Additionally, in middle-aged and older women with persistent postural-perceptual dizziness, serotonin reuptake inhibitors have been reported to have a positive impact, alleviating subjective sensations of dizziness and enhancing balance function [2]. Further, dizziness has been reported to be a prevalent withdrawal symptom associated with fluoxetine discontinuation [3,4]. However, it remains unclear whether SSRIs such as fluoxetine impact peripheral vestibular function and/or central processing of vestibular information. To investigate whether fluoxetine directly modulates ion channels involved in signal processing in the vestibular periphery, we made whole cell patch clamp recordings from afferent nerve terminals and their presynaptic hair cells in slice preparations of gerbil crista.

Vestibular hair cells are sensory receptors within the inner ear that detect acceleration and generate electrical signals that provide information to the central nervous system (CNS) regarding movement and head orientation. There are two types of hair cells, and their associated afferent neurons convey balance information to the CNS. Type I hair cells make synaptic contacts with large cup-shaped afferent nerve terminals called calyces. Type II hair cells make synaptic contacts with much smaller afferent bouton terminals and also form chemical synapses with the outer faces of calyces [5]. Each hair cell has a mechanosensitive hair bundle associated with its apical portion. Upon mechanical stimulation of the hair bundle, a transduction current flows, which generates a receptor potential and modulates synaptic transmission between the hair cell and vestibular afferent neurons. In turn, afferent fibers send action potentials to the CNS to facilitate reflexes essential for maintaining balance and stabilizing vision. Within vestibular organs, afferent firing properties vary with terminal location in the neuroepithelium, and afferents fire action potentials with variable interspike intervals [6]. Calyx afferent terminals exhibit a variety of ion channels that mediate Na^+^, K^+^, and hyperpolarization-activated conductance. Specific ion channels underlying these conductances shape afferent firing activity, determining the electrical behavior required for sensory interpretation of vestibular signals [7,8,9,10,11,12,13,14].

Fluoxetine has been reported to block a subset of voltage-dependent delayed rectifier K^+^ channels in a variety of cells [15,16,17,18]. Here, we tested its effect on ionic currents in vestibular afferent terminals. We found that micromolar concentrations of fluoxetine strongly reduced K^+^ currents in calyx terminals across the crista of the vestibular semicircular canals. In PZ cells, a rapidly activating, rapidly inactivating K^+^ current remained in the presence of fluoxetine, whereas in CZ cells, the residual K^+^ current in fluoxetine was sustained. Unexpectedly, in addition to reducing K^+^ conductances, we found that fluoxetine potently inhibited transient Na^+^ currents in central and peripheral zone calyces. We also tested fluoxetine on presynaptic type I and type II vestibular hair cells and found no effect on their K^+^ conductances.

## 2. Results

### 2.1. K^+^ Conductances in PZ and CZ Calyces

**Figure 1:**
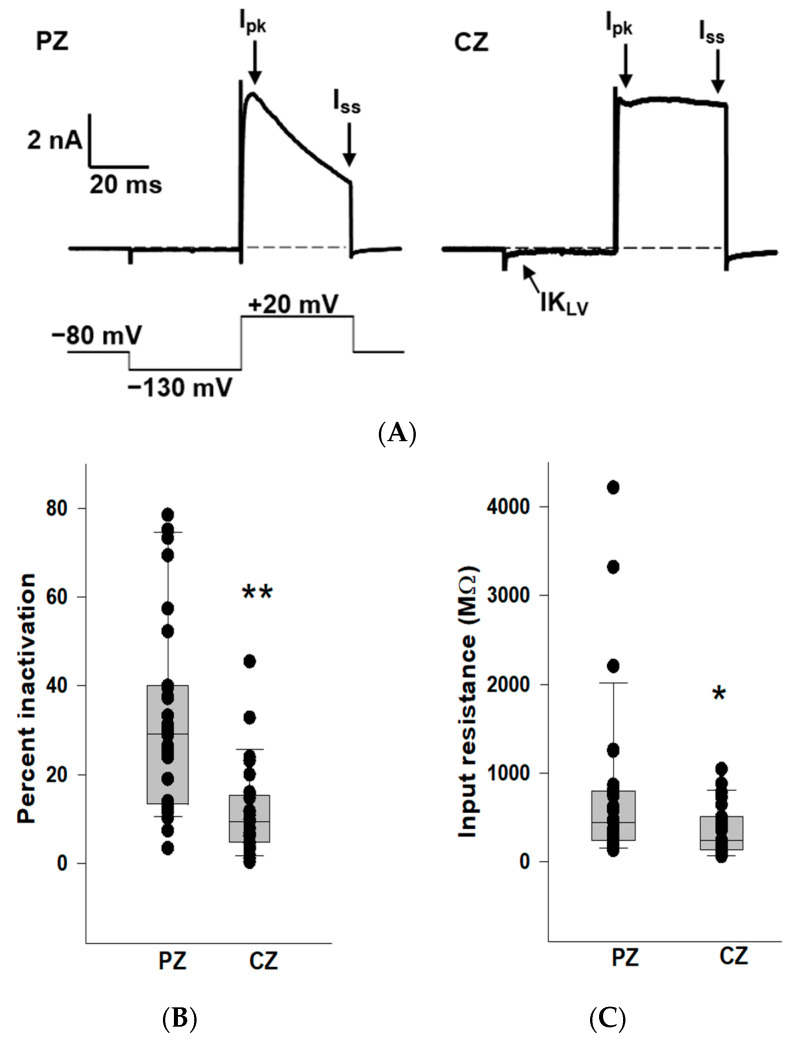
K^+^ conductances differ between PZ and CZ calyces in crista slices. (**A**) Representative K^+^ currents in PZ (left, P26, male) and CZ (right, P24, male) calyces in response to a depolarizing step following a hyperpolarization. The calyces were held at −80 mV, stepped to −130 mV, and then to +20 mV for 40 ms (voltage protocol is shown below). Dashed line in this and subsequent figures indicates zero current (0 pA). I_pk_ and I_ss_ represent current measurements at peak and end of the 40 ms pulse, respectively, and were used to assess K^+^ current inactivation. The scale bar applies to both PZ and CZ current recordings. CZ calyx shows a small low-voltage activated current (IK_LV_) that is active at the holding membrane potential of −80 mV and deactivates on stepping to the hyperpolarized potential. (**B**) Extent of K^+^ current inactivation is compared for the calyces from different zones. Each symbol indicates the percentage of inactivation measured for individual calyces from PZ and CZ. Median inactivation (line in box plot) was 29.1% in PZ calyces (*n* = 31), significantly greater than the value of 9.4% in CZ calyces (*n* = 27; *p* < 0.001, Mann–Whitney rank-sum Test) **. (**C**) Input resistance values are shown for PZ and CZ calyces. Median resistance in PZ calyces (*n* = 31) was 446.2 MΩ, which was significantly higher than the value of 245.6 MΩ in CZ calyces (*n* = 27; *p* = 0.03, Mann–Whitney rank-sum test) *.

We previously reported that outward K^+^ currents recorded from PZ calyces displayed notably greater inactivation compared to those in CZ calyces at depolarized potentials [19]. In this study, we also observed enhanced K^+^ current inactivation in PZ calyces, and we compared K^+^ current inactivation between zones (Figure 1). Patch electrodes were positioned at the base of calyx terminals to establish tight membrane seals. Following membrane breakthrough, whole-cell recordings were conducted from calyx terminals in regions identified as CZ (*n* = 27) and PZ (*n* = 31) of crista slices. The voltage protocol consisted of a conditioning pre-pulse from a holding potential of −80 mV to −130 mV followed by a depolarizing step to +20 mV. Large outward K^+^ currents at the depolarizing step were observed in both zones, but PZ calyces typically demonstrated greater inactivation in outward K^+^ currents compared to CZ calyces (Figure 1A), supporting the presence of different underlying K^+^ channels. Current amplitude was measured at the end of the step (I_ss_) and compared to peak current (I_pk_). The median inactivation was 29.1% in PZ calyces (*n* = 31), significantly greater than the value of 9.4% in CZ calyces (*n* = 27; *p* < 0.001, Mann–Whitney rank-sum test) (Figure 1B). The extent of inactivation in the PZ calyx population was quite variable between cells. Large variations in the time-dependent inactivation of K^+^ currents in isolated calyces [20,21] and in cell bodies of vestibular ganglion neurons [22,23,24] have also been reported and reflect heterogeneity in underlying K^+^ channels. In addition, CZ calyces often demonstrated more active current at the holding potential. This current deactivated on stepping to −130 mV (arrow), indicating a low-voltage-activated K^+^ current (IK_LV_), as shown in Figure 1A. We measured input resistance in calyces and found the median input resistance was 446.2 MΩ in PZ calyces (*n* = 31), significantly greater than the value of 245.6 MΩ in CZ calyces (*n* = 27; *p* = 0.03, Mann–Whitney rank-sum test) (Figure 1C). Therefore, CZ calyces had significantly lower input resistances compared to PZ calyces, consistent with the prevalence of IK_LV_ in terminals of the central zone.

### 2.2. Effects of Fluoxetine on the Outward K^+^ Current in PZ and CZ Calyces

**Figure 2:**
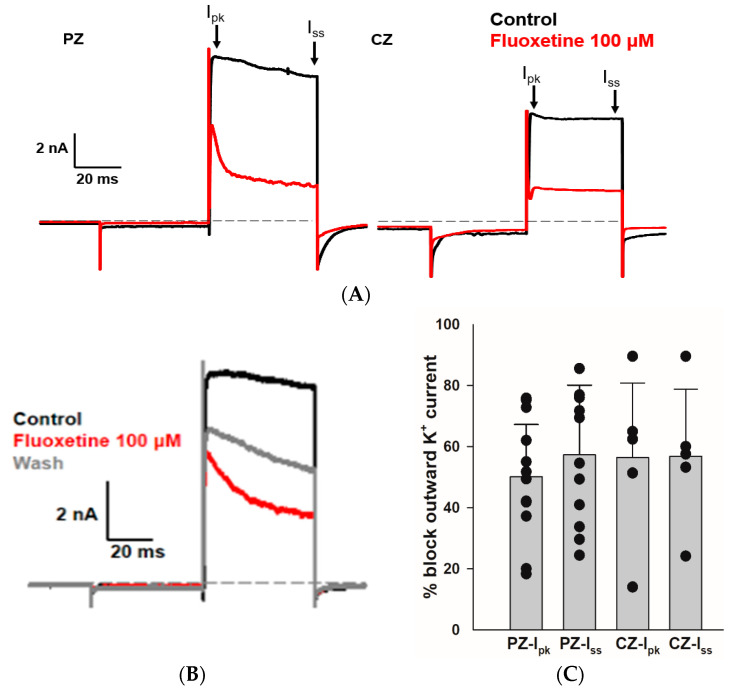
Effect of fluoxetine on K^+^ currents in PZ and CZ calyces. K^+^ currents were recorded in response to the standard voltage step to +20 mV in control conditions (black traces) and in response to 100 μM fluoxetine (red traces). (**A**) In a PZ calyx (left, P26, female), 100 μM fluoxetine reduced outward K^+^ current and revealed a rapidly activating, rapidly inactivating I_K_. In a CZ calyx (right, P28, female), fluoxetine also blocked a substantial component of outward I_K_; however, the remaining current did not inactivate. (**B**) Isolated calyx recording (female, P21, unknown zone) shows control, fluoxetine, and partial recovery of outward K^+^ current after prolonged washing with control solution (>16 min). (**C**) Fluoxetine at 100 μM blocked 50.1% of I_pk_ and 57.3% of I_ss_ in 12 PZ calyces, while in 5 CZ calyces, it blocked 56.4% of I_pk_ and 56.8% of I_ss._ Each symbol indicates the percentage of blocked outward I_K_ for individual calyces from PZ and CZ at I_pk_ and I_ss._ Bars indicate mean ± SD.

We sought to determine whether fluoxetine alters the K^+^ conductances in PZ and CZ calyces. We obtained whole-cell recordings from calyces with stable transient inward Na^+^ currents and outward K^+^ currents and subsequently perfused crista slices with 100 µM fluoxetine. Extracellular perfusion with fluoxetine typically began to reduce currents within 2 min of perfusion onset, but for measurements we allowed at least a 5 min period after the start of perfusion to ensure complete access of the drug. As shown in Figure 2, fluoxetine (100 μM) strongly reduced outward K^+^ currents in vestibular calyx terminals in both zones of the crista. Figure 2A shows the effect of fluoxetine on a PZ calyx, where it blocked a large portion of the outward K^+^ current and revealed a fluoxetine-resistant rapidly activating, rapidly inactivating A-type current. In 12 PZ calyces, fluoxetine at 100 μM blocked approximately 50.1% of I_pk_ and 57.3% of I_ss_ (Figure 2C). Fluoxetine also blocked a large portion of the non-inactivating outward K^+^ current in CZ calyces as shown for one example in Figure 2A, but it did not block the low-voltage-activated K^+^ current (IK_LV_) in response to the hyperpolarizing step from −80 mV. In five CZ calyces, fluoxetine blocked on average ~56% of I_pk_ and I_ss_, as illustrated in Figure 2C. Five calyces (three PZ, two CZ) exposed to fluoxetine were washed with normal extracellular solution over 7–10 min to try to reverse the drug effect. In one PZ calyx and an isolated calyx, some recovery of outward current was observed after prolonged washing with normal extracellular solution (isolated calyx > 16 min, Figure 2B). We measured the input resistance before and after 100 μM fluoxetine perfusion and found no significant change. In 12 PZ calyces, the median input resistance in the control solution and with 100 μM fluoxetine was 430 MΩ and 316 MΩ, respectively (*p* = 0.56, Mann–Whitney signed rank test). In five CZ calyces, the median input resistance in the control solution and with 100 μM fluoxetine was 72 MΩ and 230 MΩ, respectively (*p* = 0.54, Mann–Whitney signed rank test).

### 2.3. Effects of Fluoxetine and 4-Aminopyridine (4-AP) in PZ Calyces

**Figure 3:**
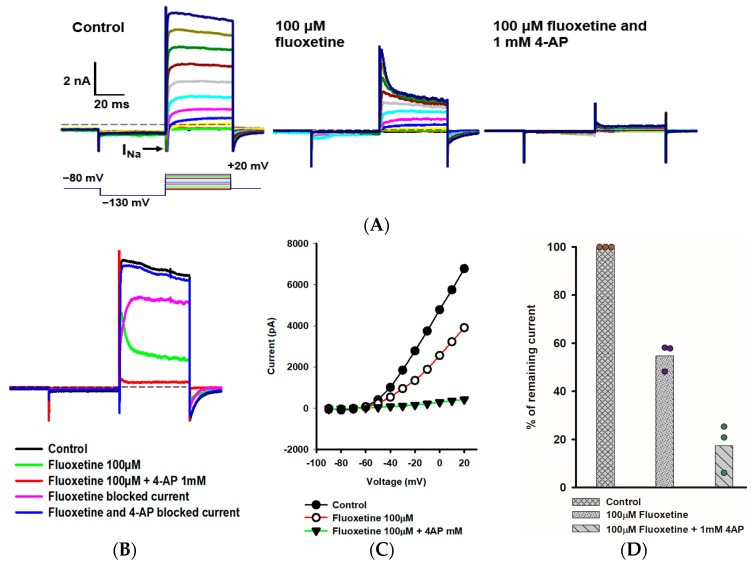
Combined effect of fluoxetine and 4-aminopyridine (4-AP) in PZ calyces. (**A**) Outward K^+^ current recorded from a PZ calyx (P23, female) in response to a range of voltage steps. From a holding potential of −80 mV, the membrane was stepped to −130 mV, and then stepped from −90 mV to +20 mV in 10-mV incrementing steps. Voltage protocol is shown below the control current (left panel). A rapid transient inward Na^+^ current (I_Na_) is seen at voltage steps between −60 mV and +10 mV. Currents in 100 μM fluoxetine are shown in the middle panel. Fluoxetine reduced outward I_K_, revealing a rapidly activating, rapidly inactivating K^+^ current at depolarized steps. Subsequently, a combination of 100 μM fluoxetine and 1 mM 4-aminopyridine (4-AP) resulted in complete block of the outward K^+^ current (right panel). (**B**) Impact of fluoxetine and fluoxetine/4-AP on the currents at the voltage step to +20 mV are shown. Control current (black), current following perfusion with 100 μM fluoxetine (green), and current following addition of 100 μM fluoxetine and 1 mM 4-AP (red) are shown. The fluoxetine-sensitive current (pink) and current blocked by 100 μM fluoxetine/1 mM 4-AP (blue) are also shown. As indicated, fluoxetine and 4-AP combined blocked almost all outward I_K_. (**C**) Current-voltage (I–V) plot illustrates the peak current measured over voltage steps for control (filled circles), fluoxetine (open circles), and fluoxetine/4-AP conditions (filled triangles). (**D**) Mean percentage of remaining current measured at +20 mV voltage step for PZ calyces (*n* = 3) was 54.7% after fluoxetine perfusion and 17.4% after combined perfusion with fluoxetine and 4-AP.

We further explored the rapidly activating and inactivating outward K^+^ current that persisted in PZ calyces following the application of 100 μM fluoxetine. Figure 3A shows the response of a PZ calyx to a series of membrane depolarizations. Fluoxetine reduced outward currents, revealing rapidly inactivating K^+^ currents at depolarized potentials. In a previous work, we showed that the K^+^ channel blocker 4-AP blocked a rapidly activating, rapidly inactivating K^+^ current in isolated calyces and broadened the width of action potentials [20]. The previously described 4-AP sensitive current resembled the A-type current remaining here in the presence of fluoxetine. We therefore tested a combination of fluoxetine and 1 mM 4-AP and saw an almost complete block of outward current as shown for a PZ calyx (Figure 3A). The currents blocked by fluoxetine and fluoxetine/4-AP combined were obtained by subtraction (Figure 3B). The fluoxetine-sensitive component was slowly activating, and did not show substantial inactivation during the voltage step to +20 mV. As anticipated, the 4-AP-sensitive current showed rapid activation and rapid inactivation properties characteristic of an A-type current (Figure 3B). The current-voltage (IV) plot in Figure 3C shows peak K^+^ currents under different conditions for a range of voltage steps for this cell. After perfusion with 100 μM fluoxetine, approximately 50% of the peak current remained, but almost all outward current was blocked in PZ calyces following perfusion with a combination of fluoxetine and 4-AP (Figure 3D).

### 2.4. Effects of Fluoxetine on Transient Na^+^ Currents in CZ and PZ Calyces

**Figure 4:**
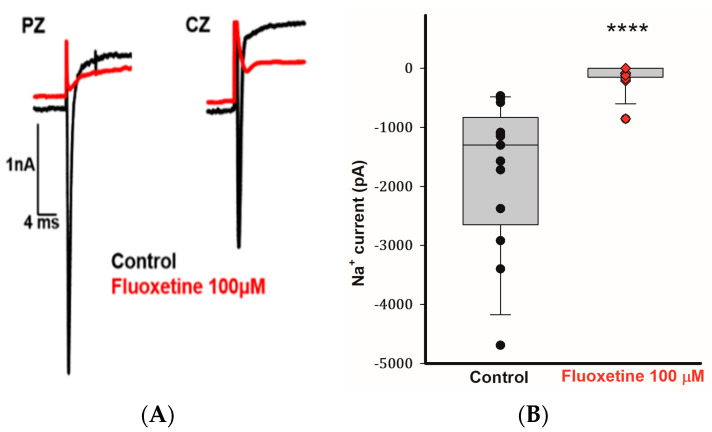
Fluoxetine (100 μM) eliminates transient Na^+^ currents in PZ and CZ calyx terminals. (**A**) Control I_Na_ (black traces) recorded at test potential steps to −40 mV following a pre-pulse to −130 mV in a PZ calyx (P23, female) and CZ calyx (P26 male). Perfusion of 100 μM fluoxetine inhibited transient inward Na^+^ currents (I_Na_) in both calyces as indicated by red traces. (**B**) Box plot demonstrates the impact of 100 μM fluoxetine on peak I_Na_ (control, black symbols) in a total of 13 cells (5 PZ calyces, 4 CZ calyces, and 4 dissociated cells) at a test step to −40 mV. Application of 100 μM fluoxetine (red symbols) effectively eliminated I_Na_. Median values of I_Na_ are shown in box plots, the control condition is significantly different after the application of 100 μM fluoxetine (*n* = 13; **** *p* < 0.0001, Wilcoxon signed-rank test).

Calyx terminals from mature (>P20) gerbil crista exhibit large transient Na^+^ currents that are blocked by tetrodotoxin (TTX) [25,26]. In addition to blocking the outward currents in CZ and PZ calyces, we observed that 100 μM fluoxetine inhibited the transient Na^+^ currents (I_Na_) in calyces from both zones. Figure 4A demonstrates the rapid inward Na^+^ current in a PZ calyx following a voltage step from −130 to −40 mV. The short-lasting inward current was completely blocked by 100 μM fluoxetine, and a similar result was seen for the rapid inward current in a CZ calyx (Figure 4A). In a total of 13 cells (5 PZ calyces, 4 CZ calyces, and 4 isolated calyces), application of 100 μM fluoxetine effectively eliminated I_Na_ (Figure 4B). Fluoxetine was previously reported to block Na^+^ currents carried by TTX-insensitive Na_v_1.5 channels associated with cardiac cells [27]. Nav1.5 channel immunoreactivity has been reported to be associated with calyx terminals in rat utricle and crista [28,29], although Na^+^ currents in mature gerbil calyx terminals are mediated in part by TTX-sensitive Na_v_1.6 channels [10]. Given the complete block of I_Na_ in calyx terminals, it appears that fluoxetine targets both Na_v_1.5 and Na_v_1.6 channels in these neurons.

### 2.5. Dose-Dependent Effects of Fluoxetine on K^+^ and Na^+^ Currents

**Figure 5:**
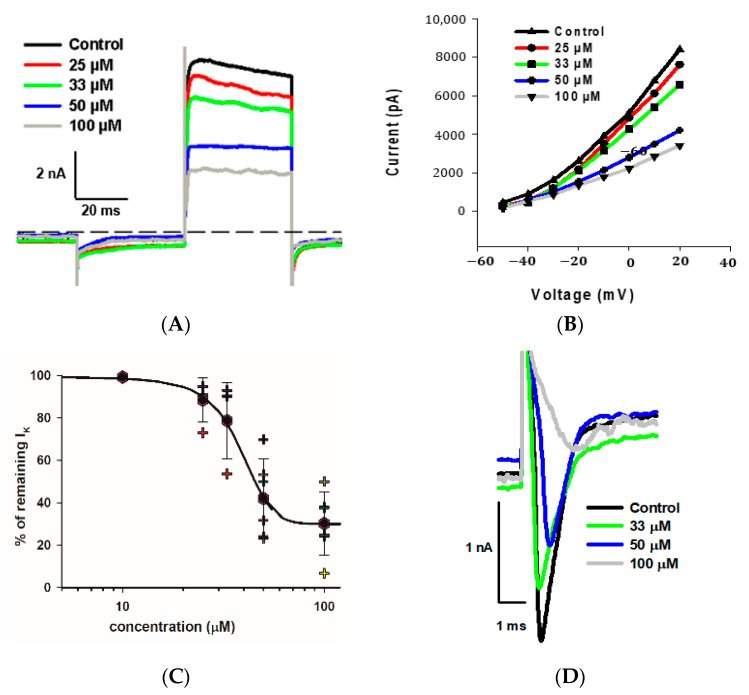

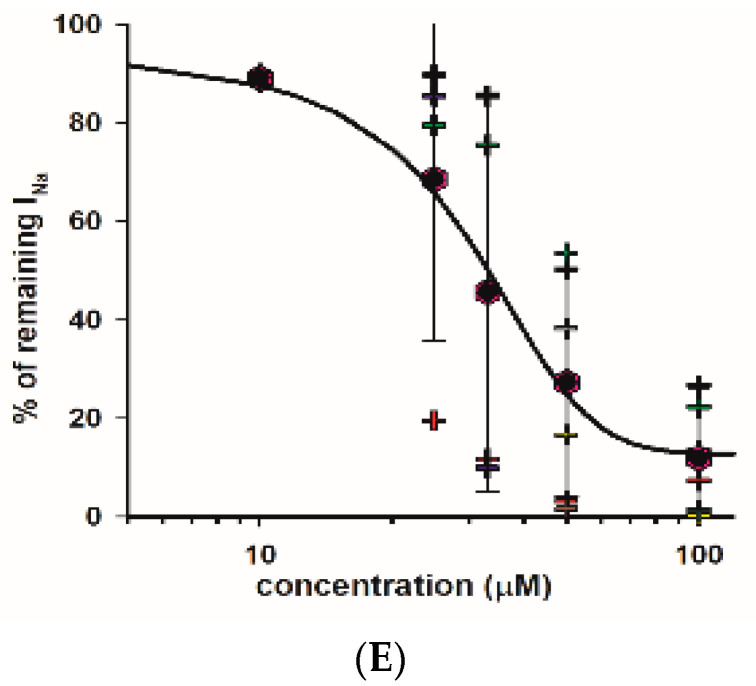
Dose dependence of blockage of K^+^ and Na^+^ currents by fluoxetine. (**A**) Superimposed outward K^+^ current recordings obtained from a CZ calyx (P30, male) in response to increasing concentrations of fluoxetine. Currents at the voltage step to +20 mV are shown. The control recording is depicted in black, while recordings for 25 μM, 33 μM, 50 μM, and 100 μM fluoxetine are shown in red, green, blue, and gray, respectively. (**B**) IV plot shows peak outward currents between −50 and +20 mV for control and increasing fluoxetine concentrations for the same cell shown in A. (**C**) Dose response for fluoxetine on K^+^ currents using various concentrations (*n* = 4 cells for PZ and 4 cells for CZ). The EC_50_, representing the concentration of fluoxetine that induces 50% of the maximum effect, was determined to be 40.1 μM (Hill coefficient −5.0). Hexagons represent mean ± SD and crosses represent individual data points. (**D**) Inward Na^+^ current recordings obtained from a CZ calyx (same as (**A**)) at the voltage step of −40 mV are superimposed for control (black trace), while those for 33 μM, 50 μM, and 100 μM fluoxetine are shown in green, blue, and gray, respectively. (**E**) Dose–response inhibitory effect of fluoxetine on inward Na^+^ currents. Hexagons represent mean ± SD and crosses represent individual data points (*n* = 4 cells for PZ and 4 cells for CZ at different concentrations). EC_50_ value for Na^+^ currents is 32.1 µM (Hill coefficient −2.6).

As indicated, we observed a large reduction in both Na^+^ and K^+^ currents in calyx terminals in response to 100 μM fluoxetine. We therefore tested a range of fluoxetine concentrations on PZ and CZ calyces to establish dose-dependence for both Na^+^ and K^+^ conductances. A representative cell from the central crista zone is shown in Figure 5A. This CZ calyx was initially perfused with solution containing 25 μM fluoxetine, and the concentration was subsequently increased in increments up to 100 μM. As expected, the outward K^+^ current decreased with increasing concentrations of fluoxetine, but the low voltage-activated K^+^ current was not blocked by concentrations up to 100 μM (Figure 5A). The IV relationship is illustrated for escalating doses of fluoxetine in Figure 5B. The dose–response effect of fluoxetine on the outward K^+^ currents at the step to +20 mV is illustrated in Figure 5C for eight cells (four PZ and four CZ calyces). The concentration of fluoxetine that produces 50% of the maximum effect (*EC_50_*) was calculated to be 40.1 μM for the outward K^+^ currents. Blockage of peak Na^+^ current also increased with increasing extracellular concentrations of fluoxetine. Figure 5D illustrates examples of superimposed current traces from a CZ calyx recorded at a voltage step to −40 mV in different concentrations of fluoxetine. The dose–response curve shows the effect on peak Na^+^ current and that the *EC_50_* for the Na^+^ currents was 32.1 μM (Figure 5E), a lower value than that obtained for K^+^ currents, indicating a more potent effect of the drug on Na^+^ channels.

### 2.6. Effect of Fluoxetine on Outward K^+^ Currents in Vestibular Hair Cells

Na^+^ and K^+^ conductances impart calyx terminals with the ability to fire action potentials. Since we observed a blockage of both conductances by micromolar concentrations of fluoxetine, an inhibition of action potential firing in vestibular afferents is predicted. However, fluoxetine might also impair inner ear sensory signals by directly interfering with ionic conductances in the presynaptic hair cells that make synapse connections with vestibular calyx afferents. In fact, fluoxetine has been reported to block K^+^ current in the outer hair cells of the cochlea [30]. We therefore tested the actions of fluoxetine on vestibular hair cells.

**Figure 6:**
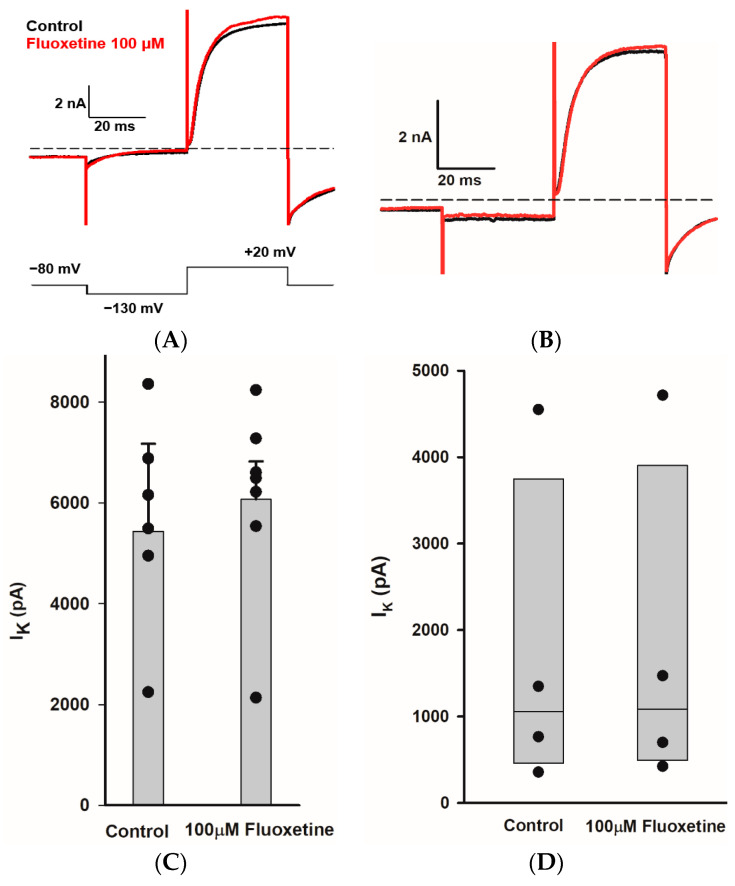
Fluoxetine does not block I_K_ in type I and type II hair cells. (**A**) K^+^ current recorded from a type I hair cell in crista slice (PZ, P22, male). Currents for control (black) and in Fluoxetine (red) are shown for the voltage step to +20 mV (voltage protocol is shown below). Application of 100 μM fluoxetine had little effect on current. (**B**) Currents recorded from a type II hair cell in crista slice (PZ, P22, female) at voltage steps to +20 mV in control (black) and 100 μM fluoxetine (red). (**C**) The mean peak outward K^+^ currents measured at +20 mV voltage step for type I hair cells. Bars indicate mean ± SD (*n* = 7, *t* = −0.2, *p* = 0.8, paired *t*-test). (**D**) Box plots represent the median peak outward K^+^ currents measured at +20 mV voltage step for type II hair cells before and after 100 μM fluoxetine perfusion (*n* = 4, *p* = 0.8, Mann–Whitney rank-sum Test).

We obtained whole cell recordings from type I and type II hair cells exhibiting distinct voltage-activated K^+^ conductances [31,32]. Type I hair cells are characterized by extremely low input resistance (<100 MΩ) conferred by a low-voltage-activated K^+^ current [33,34,35]. Recently, this K^+^ conductance has been shown to be mediated by Kv1.8 (Kcna10) K^+^ channel subunits in type I hair cells [36]. Type II hair cells do not have IK_LV_, but show smaller outward K^+^ conductances with variable inactivation. Figure 6A demonstrates the effect of perfusion of 100 μM fluoxetine on K^+^ currents in a PZ type I hair cell. Following the voltage step to −130 mV, the type I cell showed a deactivating current consistent with low-voltage-activated K^+^ current followed by a large outward current at the step to +20 mV. After several (up to 7) minutes of perfusion, there was no obvious effect of the drug on K^+^ currents evoked at the test step. For several type I hair cells tested, there was no significant difference between the mean peak outward K^+^ currents in the control and 100 μM fluoxetine conditions (Figure 6C). We also obtained whole cell recordings from type II hair cells, which showed characteristic hyperpolarization-activated current (I_h_) in response to membrane hyperpolarization below −100 mV and outward K^+^ currents at depolarizations above ~−50 mV. An example showing currents following a voltage step to +20 mV is shown for control and following perfusion with 100 μM fluoxetine for a PZ type II hair cell in Figure 6B. In type II hair cells, the mean peak outward current did not change significantly in fluoxetine (Figure 6D). Therefore, despite the pronounced blocking effect of fluoxetine on Na^+^ and K^+^ currents in calyx terminals, distinct K^+^ conductances in both types of presynaptic hair cell were unaffected by fluoxetine application.

## 3. Discussion

We studied the effects of fluoxetine, a widely used SSRI antidepressant, on whole cell conductances in afferent neurons and sensory hair cells within the crista neuroepithelium of the vestibular system of the inner ear. In addition to its use against depression, fluoxetine may also be prescribed to patients with panic disorder or obsessive-compulsive disorder. Dizziness is commonly reported following fluoxetine discontinuation, but the cause is unknown [3,4]. We found that micromolar concentrations of fluoxetine inhibited not only K^+^ currents, but also Na^+^ currents in the calyx endings of vestibular afferent neurons. Effects were specific to afferent neuron terminals, since ionic currents in type I and type II sensory hair cells were insensitive to fluoxetine at concentrations up to 100 µM. This is the first report demonstrating the inhibitory actions of fluoxetine on the electrophysiological properties of zonally specified neurons in the peripheral vestibular system.

### 3.1. Fluoxetine Blocks Outward K^+^ Currents in Vestibular Afferent Terminals

Our research revealed that the administration of fluoxetine at extracellular concentrations from 25 to 100 µM diminished voltage-dependent K^+^ currents in afferent calyx terminals. Whole cell K^+^ currents in calyces reflect ensemble currents through a combination of voltage and calcium-activated K^+^ channels [12,19,20,21,37]. Properties of vestibular sensory afferents vary with neuroepithelial location; afferents with terminals in central zones show irregular firing patterns, whereas neurons terminating in surrounding peripheral areas fire tonically with greater regularity [31]. Accumulating evidence suggests that zonal variations in afferent dendritic K^+^ channel populations contribute to these differences [9,14,19,24,28,38,39,40,41]. Here, we found that fluoxetine blocked significant voltage-activated K^+^ current in calyces terminating in both central and peripheral regions of the crista, but the characteristics of currents remaining following fluoxetine administration varied with zone. In PZ calyces, fluoxetine blocked a slowly activating and non-inactivating K^+^ current at potentials above approximately −50 mV. In the presence of fluoxetine, a rapidly activating and inactivating A-type K^+^ current was retained that was subsequently blocked by 4-aminopyridine. The A-type current is prevalent in PZ calyces and confers greater inactivation of outward K^+^ currents when compared to CZ calyces [19]. The specific K^+^ channels underpinning the A-type current are not known, but based on its kinetic profile, candidates include Kv1.4, Kv3.4, and Kv4 channels. Non-inactivating outward currents in calyces are mediated in part by members of the Kv7 (KCNQ) K^+^ channel family. There are five known Kv7 subunits (Kv7.1–5), and currents mediated by Kv7 channels often activate slowly and do not typically inactivate [42]. There is evidence for Kv7.4 and Kv7.5 subunits in vestibular afferents [19,28,38,40,43,44,45]. The Kv7 channel blockers linopirdine and XE991 were found to inhibit K^+^ current components in vestibular calyces isolated from rodent crista and utricle [21,25], and XE991 was found to block sustained outward K^+^ currents in both CZ and PZ calyces in crista slices [19]. Fluoxetine block osigmaf Kv7 channels has not been reported specifically, but fluoxetine is reported to block other voltage-dependent K^+^ channels, including delayed rectifier Kv1.1 [18,46], Kv1.3 [47], Kv2.1 [48], and also Kv3.1 channels in a variety of cell types [15,16,49]. Kv3 subunits are expressed in afferent nerve fibers innervating auditory hair cells [50,51] and in central auditory neurons [49], but their existence in vestibular afferents has not been reported.

We found that CZ calyces had a lower input resistance than PZ calyces, and this was associated with the presence of low-voltage-activated K^+^ current (IK_LV_). Previously, I_K_ sensitive to the Kv1.1/1.2 channel blockers α-dendrotoxin and dendrotoxin-K was reported to be prevalent in CZ but not PZ calyces [9,19]. Additionally, IK_LV_ is predominantly found in vestibular ganglion cell bodies associated with central neuroepithelial zones and is mediated at least partly by dendrotoxin-sensitive Kv1 channels [39,52]. IK_LV_ in CZ calyces was not blocked by fluoxetine at concentrations of up to 100 µM tested in our recordings, and input resistance was unchanged. In fact, much higher concentrations of fluoxetine (IC50 values ~0.5 mM) were required to block Kv1.1 channels expressed in oocytes [18]. High-voltage activated Kv1.3 channels are blocked by micromolar concentrations of fluoxetine [47], and could be responsible for the fluoxetine-sensitive current in calyces described here. This is supported by experiments showing that margatoxin, another Kv1.3 blocker, inhibited a slowly activating, high voltage-activated K^+^ current in both vestibular ganglion cell bodies [22,52] and calyx endings [9]. However, additional studies are needed to further pinpoint the subunit nature of the channels underlying vestibular calyx K^+^ currents sensitive to fluoxetine.

### 3.2. Fluoxetine Blocks Inward Na^+^ Currents in Vestibular Afferent Terminals

In addition to the effect on calyx K^+^ currents, we observed inhibition of voltage-dependent transient inward Na^+^ currents in centrally and peripherally located calyces with fluoxetine perfusion. We applied escalating doses of fluoxetine and found that 100 µM fluoxetine abolished Na^+^ currents. Previously, immunostaining showed evidence for Nav1.5 channels associated with the inner face of calyx terminals adjacent to type I hair cells, whereas Nav1.6 channels were reported at the heminode of dimorphic afferents in rat vestibular organs [28,29]. Electrophysiological studies have indicated that TTX blocks Na^+^ currents in mature calyx terminals (>P20), and that some of the current travels through Nav1.6 channels, based on partial block of the Na^+^ current by 4,9-anhydro-tetrodotoxin [10,26]. TTX-insensitive Nav1.5 channels may also contribute to Na^+^ current activity in immature vestibular afferent neurons [26,53]. Interestingly, Nav1.5 channels are also transiently expressed during development in rodent [26,29] and human [54] type I and type II hair cells. Although we did not test at young ages, Na^+^ currents in developing hair cells and afferents could be sensitive to blocking by fluoxetine in utero.

Fluoxetine was previously reported to block Na^+^ currents carried by TTX-insensitive cardiac Na_v_1.5 channels [27] (IC_50_ 49 µM) and Nav1.7 and Nav1.8 expressed in HEK293 cells [55] (IC_50_ values were 66 and 49 µM, respectively). The IC_50_ value of 32 µM reported here is therefore similar to values reported for other Na^+^ channel subtypes. Nav1.7 and Nav1.8 channels are expressed in sensory neurons involved in pain sensation, and SSRIs such as fluoxetine likely mediate an analgesic effect through Na^+^ channel blockade [55]. Voltage-gated Na^+^ channels produce fast, transient currents that mediate the action potential rising phase, and the availability of channels impacts action potential firing rates. Hence, inhibition of Na^+^ currents would impair Na^+^-dependent spiking in vestibular afferents and reduce transmission of signals to their central targets. Fluoxetine has been reported to suppress action potential firing in developing human cortical neurons, although the underlying mechanism has not been determined [56].

### 3.3. Fluoxetine Does Not Modulate K^+^ Currents in Vestibular Hair Cells

Vestibular epithelia in mammals contain type I and type II mechanosensory hair cells. Type I hair cells are distinguished by their amphora shape, and their basal regions are enclosed by large afferent nerve calyces forming an unusual extensive synaptic connection. Type II hair cells are more cylindrical in shape and make afferent synapses with small bouton terminals as well as the outer aspects of calyx terminals. Synaptic transmission between hair cells and afferent terminals occurs through quantal and non-quantal mechanisms [13,14,57,58,59,60,61,62,63]. We hypothesized that fluoxetine might impact vestibular signals by modulating hair cell basolateral conductances. Type I and type II hair cells demonstrate different voltage-activated K^+^ currents (reviewed in [32]). Type I hair cells display a low-voltage activated delayed rectifier K^+^ current [33,34,35] which is mediated by Kv1.8 channels [36]. Type II hair cells have A-type (Kv1.4), delayed rectifier, and calcium-activated K^+^ currents. Here, we applied fluoxetine to mature type I and type II vestibular hair cells in crista slices and demonstrated that their K^+^ conductances were unaffected by 100 µM fluoxetine. This differs from results in the cochlea, where fluoxetine blocked K^+^ current in outer hair cells [30]. The fluoxetine-sensitive current in outer hair cells was likely mediated by Kv7.4 channels, whereas mature vestibular hair cells lack the prominent presence of Kv7.4 channels [36,38,64].

### 3.4. Therapeutic Relevance

Fluoxetine is commonly prescribed to treat depression and is classified as a selective serotonin uptake inhibitor (SSRI). Although fluoxetine is generally well tolerated by patients, dizziness as a side effect is frequently reported [65]. Patients with vestibular dysfunction and anxiety have been reported to show improvements in symptoms following fluoxetine administration [1,66]. In addition, dizziness linked to SSRI withdrawal is frequently exacerbated by minor head movements, strongly suggesting an effect on the vestibular system [67,68]. Our findings indicate that micromolar concentrations of fluoxetine modulate neuronal voltage-dependent ion channels that are essential for signal transmission in the peripheral vestibular system. At a dose of 100 μM, fluoxetine completely blocked Na^+^ currents and strongly reduced K^+^ currents in vestibular afferent terminals, which would suppress action potential generation in these afferents. In vivo, such inhibition would diminish signals contributing to balance from the vestibular periphery to nuclei in the brainstem. Ion channel blockade was specific to calyx afferents, since K^+^ conductances in both type I and type II hair cells were unaffected by fluoxetine. Although plasma concentrations of fluoxetine in treated patients are ~1 µM [69], cerebral concentrations are considerably higher [70], with levels up to ~40 μM reported [71]. Fluoxetine concentrations used here (10–100 µM) are within this range, although concentrations within inner ear fluids have not been reported.

## 4. Materials and Methods

### 4.1. Crista Extraction

In accordance with protocols approved by the University of Colorado’s Institutional Animal Care and Use Committee and NIH guidelines, post-natal day (P) 20–30 Mongolian gerbils (*Meriones unguiculatus*) of both sexes were obtained from a breeding colony on location. An intraperitoneal injection of ketamine (70 mg/kg) and xylazine (5 mg/kg) mixed in sterile saline was used to anesthetize the animals. Following decapitation under deep anesthesia, the brain was removed and the inner ear exposed to reveal the membranous labyrinth. Ampullae were carefully extracted along with the ducts from the semicircular canals of the vestibular system using fine forceps and immersed immediately in a solution of Leibovitz’s L-15 medium (pH adjusted to 7.40–7.45; osmolality adjusted to 300–305 mOsmol/kg H_2_O; at room temperature ~22 °C) mixed with 0.5 mg/mL bovine serum albumin (BSA, Sigma-Aldrich, St. Louis, MO, USA) for at least 50 min before slicing.

### 4.2. Slice Preparation

Crista slicing was performed according to our previous studies [9,19]. The roof of the ampulla was cut open and the cristae carefully trimmed. Each crista was embedded in a preparation of 4% low gelling temperature agarose (2-hydroxyethylagarose, type VII; Sigma-Aldrich, St. Louis, MO, USA) dissolved in Dulbecco’s phosphate-buffered saline. A square cut block of the gel containing the crista was glued to the stage of a vibrating blade microtome (Leica VT 1200S, Leica Biosystems, Deer Park, IL, USA) using superglue. The block was inserted into the microtome holding chamber and immersed in L-15 medium. Transverse slices were cut at 100 µm, and each crista typically yielded 5–6 slices. Slices in agarose were maintained for up to 5 h in L-15 combined with 0.5 mg/mL bovine serum albumin. Prior to conducting electrophysiological recordings, a slice was moved to a recording chamber (Warner Instruments, Holliston, MA, USA) filled with L-15 solution and fastened onto the coverslip base of the chamber using a small metal weight. The slices were observed using differential interference contrast (DIC) optics through an Olympus BX51WI microscope equipped with a 40× water immersion objective lens. Neuroepithelial regions were categorized into two areas: the central zone (CZ), which covers the top apical third of the sensory epithelium of the crista, and the adjacent sloping areas, referred to as peripheral zones (PZ) [5,19,72,73]. Calyces were morphologically recognized as cup-shaped endings enveloping type I hair cells within slices. Calyx identification was confirmed in electrophysiological recordings by the detection of large transient inward Na^+^ currents in response to depolarizing steps in whole cell voltage clamp. Although rodent hair cells express Na^+^ currents during early postnatal development, they are largely absent at the postnatal ages studied here [29,74].

### 4.3. Isolated Cells Preparation

A limited number of recordings were conducted on isolated calyx terminals that remained attached to type I hair cells. Cells were mechanically separated from the cristae of P20-21 gerbils following previously outlined methods [25]. Dissociated type I hair cell/calyx pairs were permitted to settle at the base of the recording chamber before commencing recordings.

### 4.4. Electrophysiological Recordings

Capillary glass tubing (G85150T-3; Warner Instruments, Hamden, CT, USA) was placed into a micropipette puller (P-97, Sutter Instruments, San Rafael, CA, USA) and pulled to create patch pipettes. The tapered pipette ends were then heat-polished using a Narishige M830 microforge (Narishige International USA, East Meadow, NY, USA) to achieve an open tip resistance between 2.1 and 3.5 MΩ in the extracellular solution. Additionally, the tips were coated with silicone elastomer (Sylgard 184, Dow Corning, Midland, MI, USA) to minimize stray capacitance. The solution used to fill the pipettes for whole cell patch clamp recording consisted of the following components (in mM): KF (115), KCl (10), MgCl_2_ (2), NaCl (2), HEPES (10), glucose (3), ethylene glycol tetraacetic acid (EGTA) (10), and Na_2_ATP (0–2.5). The pH was adjusted to 7.4 with KOH to achieve a final K^+^ concentration of ~140 mM, and the osmolality was adjusted to 300–305 mOsm/kg H_2_O with mannitol.

The whole-cell patch clamp technique was used to establish recordings from calyx terminals, as well as type I and II hair cells, in a voltage clamp protocol. Calyces and hair cells were identified by their morphological characteristics. The recording pipette was maneuvered through the neuro-epithelial layer of the crista slice. Initially, gigaOhm seals were established on the outer surface of cells before obtaining whole-cell recordings by membrane rupture. For type I hair cell recordings, the surrounding calyx was first removed. Experiments were conducted at ambient temperature (22–24 °C). Signals were amplified utilizing a patch clamp amplifier (Axopatch 200B, Molecular Devices, Sunnyvale, CA, USA) linked to a PC via an A/D converter (Digidata 1440A, Molecular Devices) and controlled by pClamp software (version 10.7). Cells were held at a holding potential of −80 mV, and step voltage protocols were used to record Na^+^ currents and K^+^ currents. Input resistance was measured around the holding potential in voltage clamp.

To find the percentage of inactivation of K^+^ currents, current amplitude was measured at the termination of a 40 ms step to +20 mV (*I*_ss_) and compared to peak current (*I*_pk_) during the voltage step.

[(*I*_pk_ − *I*_ss_)/*I*_pk_ × 100] (1)

Data underwent online low-pass filtering at 2–5 kHz and were sampled at rates of 10–50 kHz. Liquid junction potential was computed using the Junction Potential calculator (Clampex 10.7), with corrections applied during data analysis.

### 4.5. Pharmacological Agents

Fluoxetine, obtained from Sigma-Aldrich (Chemical Sciences Company, St. Louis, MO, USA), was prepared as a 100 mM stock solution in dry DMSO, stored at −20 °C, and then diluted in L-15 to achieve final concentrations of 10, 25, 33, 50, and 100 μM on the day of experimentation. 4-aminopyridine (4-AP), obtained from Sigma-Aldrich (Chemical Sciences Company, St. Louis, MO, USA), was prepared as a 10 mM stock solution in L-15, with pH adjusted to 7.4, and stored at −20 °C, and then diluted in L-15 to achieve a final concentration of 1 mM on the day of experiment. Slices were perfused with L-15 during control conditions and drug solutions using a Gilson Minipuls 3 peristaltic pump (Gilson, Inc., Middleton, WI, USA) at a flow rate ranging from 0.5 to 1 mL/min. Perfusion with DMSO (0.1% in L-15 solution) had no effect on K^+^ currents in three cells tested.

### 4.6. Data Analysis

Analysis of the electrophysiological data was performed utilizing pClamp 10.7 (Molecular Devices) and SigmaPlot 11.0 Systat. Statistical significance was assessed using either Student’s *t*-test for normally distributed data or the non-parametric Mann–Whitney rank-sum test for data that failed the normality test. Results are presented as mean ± standard deviation (S.D.) or as medians.

## Data Availability

The data that support the findings of this study are available from the corresponding author on reasonable request.
